# Relationship between area-level socioeconomic status and health-related quality of life among cancer survivors

**DOI:** 10.1093/jncics/pkad109

**Published:** 2023-12-21

**Authors:** Claire C Conley, Heather M Derry-Vick, Jaeil Ahn, Yi Xia, Li Lin, Kristi D Graves, Wei Pan, Jane M Fall-Dickson, Bryce B Reeve, Arnold L Potosky

**Affiliations:** Department of Oncology, Georgetown University, Washington, DC, USA; Cancer Prevention Precision Control Institute, Center for Discovery and Innovation, Hackensack Meridian Health, Nutley, NJ, USA; Department of Biostatistics, Bioinformatics and Biomathematics, Georgetown University, Washington, DC, USA; Department of Biostatistics, Bioinformatics and Biomathematics, Georgetown University, Washington, DC, USA; Center for Health Measurement, Department of Population Health Sciences, Duke University School of Medicine, Durham, NC, USA; Department of Oncology, Georgetown University, Washington, DC, USA; Health Statistics and Data Science Core, Duke University School of Nursing, Durham, NC, USA; Department of Population Health Sciences, Duke University School of Medicine, Durham, NC, USA; Georgetown University School of Nursing, Georgetown University Medical Center, Washington, DC, USA; Daniel K. Inouye Graduate School of Nursing, Uniformed Services University of the Health Sciences, Bethesda, MD, USA; Center for Health Measurement, Department of Population Health Sciences, Duke University School of Medicine, Durham, NC, USA; Duke Cancer Institute, Duke University School of Medicine, Durham, NC, USA; Department of Oncology, Georgetown University, Washington, DC, USA

## Abstract

Area-level socioeconomic status (SES) impacts cancer outcomes, such as stage at diagnosis, treatments received, and mortality. However, less is known about the relationship between area-level SES and health-related quality of life (HRQOL) for cancer survivors. To assess the additive value of area-level SES data and the relative contribution of area- and individual-level SES for estimating cancer survivors’ HRQOL, we conducted a secondary analysis of data from a population-based survey study of cancer survivors (the Measuring Your Health [MY-Health] Study). Multilevel multinomial logistic regression models were used to examine the relationships between individual-level SES, area-level SES as measured by the Centers for Disease Control and Prevention’s Social Vulnerability Index, and HRQOL group membership (high, average, low, or very low HRQOL). Area-level SES did not significantly increase model estimation accuracy compared to models using only individual-level SES. However, area-level SES could be an appropriate proxy when the individual-level SES is missing.

Area-level characteristics significantly impact cancer outcomes (1-3), including health-related quality of life (HRQOL). Cancer survivors from lower socioeconomic status (SES) areas are diagnosed at more advanced disease stages and have higher mortality rates (4-9). Cancer survivors from lower SES areas also report negative effects on specific HRQOL domains such as anxiety, pain, physical well-being, and psychological well-being (10-14). These area-level differences in HRQOL remain significant even when adjusting for individual-level sociodemographic (eg, age, gender, race, ethnicity) and SES-related variables (eg, education, insurance status). Multilevel models incorporating both area- and individual-level measures are needed to yield a more comprehensive description of the complex relationship between SES and HRQOL among cancer survivors (1,15-17).

Furthermore, the relative contribution of area- and individual-level measures of SES has been debated (18-20). Area-level measures are frequently used to approximate individual-level SES when the latter data are difficult or impossible to obtain (21). Using a nationwide sample, Moss and colleagues found that area-level and individual-level SES were positively associated, but they also observed some misclassification of individual-level SES using area-level measures (22). Thus, area-level SES may not always be an appropriate proxy for individual-level SES. To our knowledge, the relative contributions of area- and individual-level SES to HRQOL have not been examined in the context of cancer survivorship.

The goals of the present study were twofold. First, we assessed the additive value of area-level SES data for estimating cancer survivors’ HRQOL. We hypothesized that models incorporating both area- and individual-level SES variables would have greater estimation accuracy than models including only individual-level SES variables. Second, we examined the relative contributions of area- and individual-level SES in estimating cancer survivors’ HRQOL. We hypothesized that models including only individual-level SES would have greater estimation accuracy than models including only area-level SES.

We conducted a secondary analysis of data from the Measuring Your Health (MY-Health) Study (23,24). Data were collected from 2010 to 2012. Adult cancer survivors (N = 5506) self-reported fatigue, pain interference, anxiety, depression, sleep disturbance, and physical, social, and cognitive function via the Patient-Reported Outcomes Measurement Information System^®^ (PROMIS^®^) short-form measures 6 to 13 months postdiagnosis (25). Given the high correlations among the 8 PROMIS HRQOL domains (23,26), we elected to examine them as a profile of symptoms/functional deficits, rather than individual domains. As previously described (23), latent profile analysis identified 4 distinct groups of survivors with similar mean scores across the 8 HRQOL domains (27-29). Thus, our primary outcome of interest was membership in 1 of 4 HRQOL groups: high, average, low, or very low HRQOL. To examine possible differences in the contribution of area-level SES by HRQOL domain, we also conducted sensitivity analyses, modeling the 8 domains as separate outcomes.

Previously published analyses demonstrated differences in HRQOL group membership by self-reported race, ethnicity, and country of origin (23), adjusting for individual-level clinical and demographic characteristics, including SES (ie, education, insurance status, and financial well-being). The present analyses build on these models by incorporating area-level SES. Specifically, we curated area-level SES data based on the census tract of residence at diagnosis, which was the smallest geographic area for which our SES measure was available. Census tract information was available for 99.5% (5481/5506) of participants. Area-level SES was assessed using the 2010 Social Vulnerability Index (SVI) (30,31), a composite measure including key social determinants of health variables, which has been frequently used in studies across the cancer control continuum (4). The SVI summarizes data from the United States Census and the American Community Survey into 4 themes: (1) SES, (2) household composition/disability, (3) minority status/language, and (4) housing type/transportation. Our analysis focused on Theme 1 (SES), which is a composite measure incorporating persons below the poverty line, persons without a high school diploma, unemployment rates, and per capita income. Census tracts are scored from 0 to 100, with higher scores denoting poorer conditions and more social vulnerability. SVI scores were further explored using categorization into tertiles (low, medium, and high vulnerability).

For our primary outcome of interest (HRQOL group membership), multilevel multinomial logistic regression models (MLMLM) were conducted to examine the relationships between individual-level variables, area-level SES, and HRQOL group membership; census tract was included as a random effect to describe clustering of participants within census tracts. For MLMLM, model performance was assessed via model estimation accuracy. MLMLM generates an estimate for each participant’s HRQOL group on the basis of the included independent variables. The model-generated estimate can then be compared with the participant’s actual HRQOL group. Estimation accuracy is calculated by dividing the number of correct estimates by the total number of estimates (or the number of total participants). For sensitivity analyses, multilevel linear regression models were conducted to examine the relationships between individual-level variables, area-level SES, and each PROMIS domain. For linear models, model fit was assessed via adjusted *R*-square, root mean square error (RMSE), and Akaike information criterion (AIC). Data were assumed to be missing at random, and we used all available data to estimate the models. All tests were 2-sided (*P *<* *.05). Analyses were conducted using SAS (version 9.4).

Results of MLMLM are summarized in Table 1. Our starting point was the final model described by Reeve et al. (23). The model estimation accuracy of this initial model (Model 1) was 53.73%. When area-level SVI Theme 1 (SES) was combined with the individual-level variables in Model 2, model estimation accuracy marginally increased to 54.15%. The difference in accuracy between Model 1 and Model 2 was not statistically significant (*P *=* *.726; see Table 2). However, SVI Theme 1 had statistically significant relationships with HRQOL group membership (*P *<* *.001). The relationships between SVI Theme 1 tertiles and individual-level variables are presented in Supplementary Table 1 (available online).

**Table 1.** Results of multilevel multinomial logistic regression models (MLMLM) examining the relationship between individual-level variables and/or Social Vulnerability Index (SVI) and health-related quality of life (HRQOL) group membership

Model	**Estimation accuracy** ^e^	**Kapp*a*** ^f^	**SVI Theme 1 *P*-value** ^g^
**Continuous SVI Theme 1**			
M1: Individual-level variables from Reeve et al. (23)^a^	0.5373	0.3440	—^h^
${M2\text{:}\;M1+\text{SVI}}^{b}$	0.5415	0.3504	.007^i^
${M3\text{:}\;M1-\text{individual SES}}^{c}$	0.5230	0.3226	—^h^
${M4\text{:}\;M1+\text{SVI}—\text{individual SES}}^{d}$	0.5310	0.3345	<.0001^i^
**Tertile SVI Theme 1 (low, medium, high)**			
M1: Individual-level variables from Reeve et al. (23)^a^	0.5373	0.3440	—^h^
${M2\text{:}\;M1+\text{SVI}}^{b}$	0.5429	0.3522	.114
${M3\text{:}\;M1-\text{individual SES}}^{c}$	0.5230	0.3226	—^h^
${M4\text{:}\;M1+\text{SVI}—\text{individual SES}}^{d}$	0.5233	0.3237	.0001

a Model 1 (M1) includes age at cancer diagnosis, race and ethnicity group, born in the United States, survey language, education, marital status, employment status, insurance status, smoking status, cancer type, cancer stage, treatment modality, heart-related condition, lung-related condition, mental-health-related condition, sleep disturbance condition, companionship, financial well-being, and spiritual well-being (see Reeve et al. [23]). SES = socioeconomic status.

b Model 2 (M2) includes variables in M1, SVI Theme 1, and area-level random effects.

c Model 3 (M3) includes variables in M1 except education, insurance status, and financial well-being.

d Model 4 (M4) includes variables in M1 except education, insurance status, and financial well-being; SVI Theme 1; and area-level random effects.

e Estimation accuracy was computed as correctly estimated HRQOL groups/(correctly + incorrectly estimated HRQOL groups).

f The kappa coefficient is a measurement of agreement (0 < kappa < 1). The higher value indicates the better agreement between the observed and estimated HRQOL groups.

g When SVI Theme 1 was included, the likelihood ratio test was used to compute *P* value.

h No value is provided as SVI Theme 1 was not included in this model.

i For a 1-unit (%) increase in SVI Theme 1, the estimated odds ratio (95% confidence interval) of (1) average vs high HRQOL groups is 1.054 (1.007–1.104, *P *=* *.024); (2) low vs high HRQOL is 1.012 (0.974–1.051, *P *=* *.537); (3) very low vs high HRQOL is 1.113 (1.042–1.189, *P *=* *.001); (4) low vs average HRQOL is 0.96 (0.924–0.997, *P *= .036); (5) very low vs average HRQOL is 1.056 (0.998–1.117, *P *=* *.058); and (6) very low vs low HRQOL is 1.1(1.036–1.168, *P *=* *.002).

j For a 1-unit (%) increase in SVI Theme 1, the estimated odds ratio (95% confidence interval) of (1) average v. high HRQOL groups is 1.092 (1.045–1.140, *P *<* *.001); (2) low vs high HRQOL is 1.03 (0.993–1.069, *P *=* *.112); (3) very low vs high HRQOL is 1.186 (1.113–1.262, *P *<* *.001); (4) low vs average HRQOL is 0.944 (0.91–0.978, *P *=* *.002); (5) very low vs average HRQOL is 1.086 (1.029–1.146, *P *=* *.003); and (6) very low vs low HRQOL is 1.151 (1.087–1.218, *P *<* *.001).

**Table 2.** Tests of pairwise differences in model estimated accuracy using Fisher exact test^a^

	*P*		
Model	M2^c^	M3^d^	M4^e^
**Continuous SVI Theme 1**			
M1^b^	0.7263	0.2301	0.5961
M2^c^	—^f^	0.1211	0.3789
M3^d^	—	—	0.5029
**Tertile SVI Theme 1 (low, medium, high)**			
M1^b^	0.6384	0.2301	0.2380
M2^c^	—	0.0949	0.0989
M3^d^	—	—	0.9761

a These pairwise tests do not adjust for multiplicity that arose when pairing 2 models.

b Model 1 (M1) includes age at cancer diagnosis, race and ethnicity group, born in the United States, survey language, education, marital status, employment status, insurance status, smoking status, cancer type, cancer stage, treatment modality, heart-related condition, lung-related condition, mental-health-related condition, sleep disturbance condition, companionship, financial well-being, and spiritual well-being (see Reeve et al. [23]).

c Model 2 (M2) includes variables in M1, Social Vulnerability Index (SVI) Theme 1, and area-level random effects.

d Model 3 (M3) includes variables in M1 except education, insurance status, and financial well-being.

e Model 4 (M4) includes variables in M1 except education, insurance status, and financial well-being; SVI Theme 1; and area-level random effects.

f No value is given in empty cells because this table presents pairwise comparisons and values would be redundant with those already displayed.

We next examined the estimation accuracy of a model excluding self-reported education, insurance status, and financial well-being (Model 3) compared with Model 1. The model estimation accuracy of Model 3 was 52.30%. Thus, removing individual-level SES variables resulted in a 1.43% decrease in estimation accuracy. However, the difference in accuracy between Model 1 and Model 3 was not statistically significant (*P *=* *.230). Our final model (Model 4) examined whether SVI Theme 1 in the absence of individual-level SES variables could recover the 1.43% deficit in Model 3 and thus be used as a proxy for individual-level SES variables. The estimation accuracy of Model 4 was improved to 53.10% but was still less accurate than Model 1 (53.73%). The difference in accuracy between Model 1 and Model 4 was not statistically significant (*P *=* *.596). In Model 4, SVI Theme 1 was again significantly related to HRQOL group membership (*P *<* *.001). The findings were similar when we used SVI Theme 1 tertile categories (see Supplementary Table 2, available online). Additional model fit statistics are provided in Supplementary Table 3 (available online).

Sensitivity analyses examined the value of SVI Theme 1 in estimating each PROMIS domain individually (see Supplementary Table 4, available online). The results were similar to the primary analyses. Specifically, the addition of SVI Theme 1 (Model 2) improved the model fit across all 8 domains in terms of the adjusted *R*-square and RMSE. However, the performance of Model 2 (vs Model 1) was slightly worse according to AIC. In Model 2, SVI Theme 1 was significantly associated (in the expected directions) with fatigue, pain interference, depression, and physical and social function (all *P *<* *.025). The addition of SVI Theme 1 in the absence of individual-level SES variables (Model 4) resulted in improved fit (vs Model 3) across all 8 domains in terms of the adjusted R-square, RMSE, and AIC. In Model 4, SVI Theme 1 was significantly associated (in the expected directions) with all HRQOL domains (all *P *<* *.028) except for cognitive function (*P *=* *.242).

In summary, adding area-level SES (SVI Theme 1) did not significantly increase the estimation accuracy of our initial model using only individual-level SES measures. However, if individual-level measures of SES were not available, area-level SES could increase the degree of accuracy of models examining HRQOL among cancer survivors. Although models with area-level SES achieved slightly lower accuracy than those with individual-level SES measures, the relative ease of collecting and curating area-level data may outweigh the limitations (32).

Results should be interpreted cautiously in light of limitations. The SVI measures vulnerability to disaster (33) and does not capture all components of SES relevant to cancer survivorship (eg, insurance status, access/proximity to care). Thus, other area-level measures might perform differently in models examining HRQOL among cancer survivors (34,35). Future studies might incorporate other SES indicators (eg, social class, total wealth) (36). Additionally, the majority of our sample (93%) resided in census tracts classified as “metropolitan areas” (37). However, composite measures of area-level vulnerability such as the SVI may have differential utility and/or interpretations in urban and rural settings (38). Future research is needed to examine the value of SVI Theme 1 in estimating HRQOL across different geographic regions.

Despite increasing recognition of the impact of area-level SES on cancer outcomes, our results did not demonstrate the additive value of area-level SES, above and beyond individual-level SES, in estimating HRQOL among cancer survivors. However, area-level SES might be an appropriate proxy when individual-level SES are not available.

## Supplementary Material

pkad109_Supplementary_Data

## Data Availability

The data analyzed in this study are available with permission from the Harvard Dataverse for HealthMeasures at: https://doi.org/10.7910/DVN/XD1A6B.
