# Supplementary material for: Relationship between area-level socioeconomic status and health-related quality of life among cancer survivors
Source: JNCI Cancer Spectr. 2023 Dec 21;8(1):pkad109. doi: 10.1093/jncics/pkad109 (PMC10868382; doi:10.1093/jncics/pkad109)
Supplement: pkad109_Supplementary_Data [file pkad109_supplementary_data.pdf]

Supplementary Table 1. Relationship between SVI Theme 1 tertiles (low, medium, and high-vulnerability) and individual-level variables (N=5,341).

	Low (n=2044)	Medium (n=1606)	High (n=1691)	*P*-value
Age at diagnosis (median, IQR)	62 (51, 70)	61 (51, 69)	61 (51, 69)	0.2330
Race and Ethnicity (*n*, %)				<0.0001
Non-Hispanic White	1,095 (54)	710 (45)	391 (23)	
Non-Hispanic Black	197 (10)	276 (17)	624 (37)	
Chinese	182 (9)	82 (5)	36 (2)	
Other Asian[a]	177 (9)	91 (6)	54 (3)	
Filipino	107 (5)	112 (7)	64 (4)	
Other Race[b]	100 (5)	123 (8)	183 (11)	
Mexican	81 (4)	126 (8)	202 (12)	
Other Hispanic[c]	62 (3)	48 (3)	83 (5)	
Caribbean[d]	30 (1)	27 (2)	49 (3)	
Born in the US (*n*, %)				0.2802
No	578 (28)	438 (27)	497 (30)	
Yes	1,455 (72)	1,156 (73)	1,161 (70)	
Survey language (*n*, %)				<0.0001
English	1,932 (95)	1,482 (92)	1,460 (86)	
Spanish	50 (2)	83 (5)	205 (12)	
Mandarin Chinese	62 (3)	41 (3)	26 (2)	
Education (*n*, %)				<0.0001
< High school degree	140 (7)	262 (17)	495 (30)	
High school degree/some college	927 (46)	902 (57)	936 (56)	
College/graduate degree	967 (48)	414 (26)	228 (14)	
Marital status (*n*, %)				<0.0001
Never married/separated/divorced/widowed	574 (28)	604 (38)	801 (48)	
Married/living with partner	1,453 (72)	982 (62)	872 (52)	
Employment (*n*, %)				<0.0001
Working	1,059 (52)	693 (44)	629 (38)	
Not working	970 (48)	891 (56)	1,021 (62)	
Insurance (*n*, %)				<0.0001
Private/Private + Government	1,625 (82)	1,074 (71)	828 (53)	
Government/No Insurance	359 (18)	448 (29)	730 (47)	
Smoking status (*n*, %)				<0.0001
Current smoker	112 (6)	168 (11)	256 (15)	
Former smoker	729 (36)	595 (37)	505 (30)	
Never smoker	1,181 (58)	829 (52)	912 (55)	
Cancer type (*n*, %)				<0.0001
Breast	678 (33)	503 (31)	450 (27)	
Cervix	50 (2)	41 (3)	53 (3)	
Colorectal	311 (15)	258 (16)	337 (20)	
Lung	233 (11)	222 (14)	221 (13)	
NHL	180 (9)	142 (9)	118 (7)	
Prostate	453 (22)	331 (21)	372 (22)	
Uterus	139 (7)	109 (7)	140 (8)	
Cancer stage (*n*, %)				<0.0001
In situ/Stage I	848 (43)	576 (37)	552 (34)	
Stage II	622 (31)	520 (33)	543 (33)	
Stage III	302 (15)	283 (18)	312 (19)	
Stage IV	205 (10)	177 (11)	220 (14)	
Treatment modality				<0.0001
Local only (surgery and/or RT)	612 (33)	415 (30)	371 (26)	
Surgery and CT (with or without HT)	300 (16)	253 (18)	306 (21)	
RT and CT (with or without HT)	99 (5)	121 (9)	128 (9)	
Surgery, RT, CT (with or without HT)	331 (18)	272 (20)	256 (18)	
Surgery and/or RT, with HT alone	203 (11)	112 (8)	103 (7)	
Systemic only (CT and/or HT)	158 (9)	120 (9)	134 (9)	
No therapy done	124 (7)	101 (7)	134 (9)	
Heart-related condition: Yes (*n*, %)	207 (10)	218 (14)	271 (17)	<0.0001
Lung-related condition: Yes (*n*, %)	361 (18)	334 (22)	362 (22)	0.0033
Mental health condition: Yes (*n*, %)	474 (24)	429 (28)	501 (31)	<0.0001
Sleep disturbance condition: Yes (*n*, %)	240 (12)	251 (16)	283 (17)	<0.0001

Companionship (*n*, %)				<0.0001
Never/Rarely	143 (7)	181 (11)	265 (16)	
Sometimes	278 (14)	245 (15)	363 (22)	
Often/Always	1,587 (79)	1,157 (73)	1,025 (62)	
Financial well-being (median, IQR)	62.5 (43.8, 81.3)	56.3 (37.5, 75.0)	56.3 (37.5, 75.0)	<0.0001
Spiritual well-being (median, IQR)	39 (31, 45)	39 (31, 45)	40 (32, 45)	0.0130

[a] Other Asian included Japan, Korea, Vietnam, India, and other Asian countries.
[b] Other Race included Native American Indian, Alaska Natives, Pacific islands, and other or multiple races.
[c] Other Hispanic group included a mix of countries with sample sizes less than 25, including: Argentina, Brazil, Chile, Colombia, Ecuador, El Salvador, Guatemala, Honduras, Nicaragua and Peru.
[d] Caribbean included Puerto Rico, Cuba, and Dominican Republic.

Supplementary Table 2. Multi-level multinomial logistic regression models (MLMLM) examining the relationship between tertile SVI and HRQOL group membership.

SVI Theme 1 Tertile (# Denotes Reference Category)	Comparator v. Reference HRQOL Profile	Adjusted Odds Ratio (95% Wald CI)	*P*-value
**Model 2 (M2)[a]**			
High vulnerability v. Low vulnerability#	Average v. High	1.392 (1.014, 1.912)	0.0408
	Low v. High	1.073 (0.824, 1.398)	0.6012
	Very Low v. High	1.884 (1.190, 2.984)	0.0069
	Low v. Average	0.771 (0.593, 1.001)	0.0510
	Very Low v. Average	1.353 (0.913, 2.005)	0.1310
	Very Low v. Low	1.757 (1.156, 2.669)	0.0083
Medium vulnerability v. Low vulnerability#	Average v. High	1.198 (0.911, 1.574)	0.1954
	Low v. High	0.981 (0.785, 1.226)	0.8639
	Very Low v. High	1.308 (0.854, 2.004)	0.2167
	Low v. Average	0.819 (0.650, 1.033)	0.0910
	Very Low v. Average	1.092 (0.749, 1.593)	0.6460
	Very Low v. Low	1.333 (0.897, 1.982)	0.1552
**Model 4 (M4)[b]**			
High vulnerability v. Low vulnerability#	Average v. High	1.747 (1.290, 2.367)	0.0003
	Low v. High	1.207 (0.934, 1.560)	0.1496
	Very Low v. High	2.883 (1.858, 4.474)	<0.0001
	Low v. Average	0.691 (0.537, 0.888)	0.0038
	Very Low v. Average	1.650 (1.131, 2.407)	0.0093
	Very Low v. Low	2.389 (1.602, 3.563)	<0.0001
Medium vulnerability v. Low vulnerability#	Average v. High	1.388 (1.064, 1.810)	0.0157
	Low v. High	1.047 (0.841, 1.303)	0.6816
	Very Low v. High	1.722 (1.137, 2.607)	0.0103
	Low v. Average	0.755 (0.602, 0.946)	0.0148
	Very Low v. Average	1.241 (0.858, 1.795)	0.2523
	Very Low v. Low	1.644 (1.117, 2.420)	0.0117

[a] Model 2 (M2) includes variables from Reeve et al. (23), SVI Theme 1, and area-level random effects.

[b] Model 4 (M4) includes variables from Reeve et al. (23) except education, insurance status, and financial well-being; SVI Theme 1; and area-level random effects.

Supplementary Table 3. Model statistics by class.

Model with SVI percentile (continuous)		High HRQOL	Average HRQOL	Low HRQOL	Very Low HRQOL	Model with SVI tertile (categorical)		High HRQOL	Average HRQOL	Low HRQOL	Very Low HRQOL
M1[a]	Sensitivity	0.5971	0.4895	0.5604	0.4205	M1	Sensitivity	0.5971	0.4895	0.5604	0.4205
	Specificity	0.8645	0.8245	0.6736	0.9712		Specificity	0.8645	0.8245	0.6736	0.9712
	Pos Pred Value	0.6243	0.5087	0.4861	0.6193		Pos Pred Value	0.6243	0.5087	0.4861	0.6193
	Neg Pred Value	0.8505	0.8131	0.7355	0.9378		Neg Pred Value	0.8505	0.8131	0.7355	0.9378
	Prevalence	0.2739	0.2708	0.3552	0.1001		Prevalence	0.2739	0.2708	0.3552	0.1001
	Detection Rate	0.1635	0.1325	0.1991	0.0421		Detection Rate	0.1635	0.1325	0.1991	0.0421
	Detection Prevalence	0.2619	0.2605	0.4096	0.0680		Detection Prevalence	0.2619	0.2605	0.4096	0.0680
	Balanced Accuracy	0.7308	0.6570	0.6170	0.6959		Balanced Accuracy	0.7308	0.6570	0.6170	0.6959
M2[b]	Sensitivity	0.6044	0.5000	0.5572	0.4261	M2	Sensitivity	0.6033	0.5011	0.5604	0.4290
	Specificity	0.8645	0.8225	0.6802	0.9728		Specificity	0.8649	0.8241	0.6793	0.9734
	Pos Pred Value	0.6272	0.5113	0.4898	0.6356		Pos Pred Value	0.6274	0.5140	0.4905	0.6426
	Neg Pred Value	0.8528	0.8159	0.7360	0.9384		Neg Pred Value	0.8525	0.8165	0.7372	0.9387
	Prevalence	0.2739	0.2708	0.3552	0.1001		Prevalence	0.2739	0.2708	0.3552	0.1001
	Detection Rate	0.1655	0.1354	0.1980	0.0427		Detection Rate	0.1652	0.1357	0.1991	0.0430
	Detection Prevalence	0.2639	0.2648	0.4042	0.0671		Detection Prevalence	0.2634	0.2639	0.4059	0.0668
	Balanced Accuracy	0.7344	0.6613	0.6187	0.6995		Balanced Accuracy	0.7341	0.6626	0.6199	0.7012
M3[c]	Sensitivity	0.5784	0.4769	0.5532	0.3892	M3	Sensitivity	0.5784	0.4767	0.5532	0.3892
	Specificity	0.8566	0.8222	0.6603	0.9731		Specificity	0.8566	0.8222	0.6603	0.9731
	Pos Pred Value	0.6035	0.4989	0.4730	0.6171		Pos Pred Value	0.6035	0.4989	0.4730	0.6171
	Neg Pred Value	0.8434	0.8089	0.7285	0.9347		Neg Pred Value	0.8434	0.8089	0.7285	0.9347
	Prevalence	0.2739	0.2708	0.3552	0.1001		Prevalence	0.2739	0.2708	0.3552	0.1001
	Detection Rate	0.1584	0.1291	0.1965	0.0390		Detection Rate	0.1584	0.1291	0.1965	0.0390
	Detection Prevalence	0.2625	0.2588	0.4155	0.0631		Detection Prevalence	0.2625	0.2588	0.4155	0.0631
	Balanced Accuracy	0.7175	0.6495	0.6068	0.6812		Balanced Accuracy	0.7175	0.6495	0.6068	0.6812
M4[d]	Sensitivity	0.5826	0.4874	0.5580	0.4119	M4	Sensitivity	0.5815	0.4727	0.5500	0.4063
	Specificity	0.8562	0.8284	0.6665	0.9728		Specificity	0.8539	0.8272	0.6608	0.9712
	Pos Pred Value	0.6045	0.5133	0.4797	0.6277		Pos Pred Value	0.6002	0.5039	0.4718	0.6111
	Neg Pred Value	0.8447	0.8132	0.7324	0.9370		Neg Pred Value	0.8440	0.8086	0.7272	0.9363
	Prevalence	0.2739	0.2708	0.3552	0.1001		Prevalence	0.2739	0.2708	0.3552	0.1001
	Detection Rate	0.1596	0.1320	0.1982	0.0412		Detection Rate	0.1593	0.1280	0.1954	0.0467
	Detection Prevalence	0.2639	0.2571	0.4133	0.0657		Detection Prevalence	0.2654	0.2540	0.4141	0.0666
	Balanced Accuracy	0.7194	0.6579	0.6123	0.6924		Balanced Accuracy	0.7177	0.6500	0.6054	0.6887

[a] Model 1 (M1) includes age at cancer diagnosis, race and ethnicity group, born in the United States, survey language, education, marital status, employment status, insurance status, smoking status, cancer type, cancer stage, treatment modality, heart-related condition, lung-related condition, mental health-related condition, sleep disturbance condition, companionship, financial well-being, and spiritual well-being (see Reeve et al. (23)).

[b] Model 2 (M2) includes variables in M1, SVI Theme 1, and area-level random effects.

[c] Model 3 (M3) includes variables in M1 except education, insurance status, and financial well-being.

[d] Model 4 (M4) includes variables in M1 except education, insurance status, and financial well-being; SVI Theme 1; and area-level random effects.

Supplementary Table 4. Results of sensitivity analyses examining relationship between SVI Theme 1 and PROMIS® domains.

					SVI Theme 1	
**Model**	**PROMIS® Domain**	**Adjusted R^2**	**RMSE**	**AIC**	**Parameter Estimate (β)**	***P*-value**
M1[a]	Physical function	0.3954	7.4714	24185.2880	--	--
	Social function	0.3558	8.4771	25086.8179	--	--
	Cognitive function	0.3080	9.4507	25814.4722	--	--
	Sleep disturbance	0.2562	8.7182	25262.4620	--	--
	Anxiety	0.4217	8.1433	24712.9982	--	--
	Depression	0.4861	7.4317	24065.7467	--	--
	Fatigue	0.3412	8.7335	25289.1800	--	--
	Pain	0.2629	9.1493	25623.2548	--	--
M2[b]	Physical function	0.4028	7.4512	24192.6538	-0.2249	0.0000
	Social function	0.3623	8.4657	25092.7723	-0.1802	0.0022
	Cognitive function	0.3136	9.2684	25819.0567	-0.0191	0.7733
	Sleep disturbance	0.2634	8.5322	25273.3134	0.0645	0.2911
	Anxiety	0.4264	7.8258	24723.3821	0.1123	0.0503
	Depression	0.4898	7.1150	24082.3498	0.1174	0.0251
	Fatigue	0.3471	8.6453	25296.5002	0.1392	0.0224
	Pain	0.2734	9.1222	25611.3913	0.2885	0.0000
M3[c]	Physical function	0.3651	7.6607	24353.1686	--	--
	Social function	0.3403	8.5833	25166.3989	--	--
	Cognitive function	0.2979	9.5253	25861.6595	--	--
	Sleep disturbance	0.2489	8.7658	25292.7507	--	--
	Anxiety	0.4038	8.2735	24816.0887	--	--
	Depression	0.4731	7.5290	24148.8257	--	--
	Fatigue	0.3229	8.8586	25381.1254	--	--
	Pain	0.2387	9.3039	25733.0910	--	--
M4[d]	Physical function	0.3785	7.6042	24316.2520	-0.3663	0.0000
	Social function	0.3489	8.5075	25150.2604	-0.2667	0.0000
	Cognitive function	0.3033	9.3607	25856.8668	-0.0751	0.2415
	Sleep disturbance	0.2564	8.5804	25291.2000	0.1298	0.0278
	Anxiety	0.4105	7.9228	24803.5593	0.2197	0.0001
	Depression	0.4784	7.1728	24143.4380	0.1964	0.0001
	Fatigue	0.3308	8.7252	25367.8297	0.2382	0.0001
	Pain	0.2547	9.2192	25685.7558	0.4238	0.0000
	Physical function	0.3785	7.6042	24316.2520	-0.3663	0.0000

[a] Model 1 (M1) includes age at cancer diagnosis, race and ethnicity group, born in the United States, survey language, education, marital status, employment status, insurance status, smoking status, cancer type, cancer stage, treatment modality, heart-related condition, lung-related condition, mental health-related condition, sleep disturbance condition, companionship, financial well-being, and spiritual well-being (see Reeve et al. (23)).
[b] Model 2 (M2) includes variables in M1, SVI Theme 1, and area-level random effects.
[c] Model 3 (M3) includes variables in M1 except education, insurance status, and financial well-being.
[d] Model 4 (M4) includes variables in M1 except education, insurance status, and financial well-being; SVI Theme 1; and area-level random effects.
AIC = Akaike information criterion; RMSE = root mean square error
